# Atypical presentation of mpox in Irrua environs: a case report

**DOI:** 10.1186/s13256-023-04225-0

**Published:** 2023-11-26

**Authors:** S. O. Oiwoh, E. A. Tobin, D. A. Asogun, C. O. Erameh, K. O. Iraoyah, J. Okoeguale, R. A. Eifediyi, S. O. Samuel, T. A. T. Salami, S. A. Okogbenin

**Affiliations:** 1 Dermatology and Venereology Unit, Department of Internal Medicine, Irrua Specialist Teaching Hospital, Irrua, Edo State Nigeria; 2 Pan-African Network For Rapid Research, Response, Relief and Preparedness for Infectious Disease Epidemics (PANDORA-ID-NET), Institute of Viral Hemorrhagic Fevers and Emerging Pathogens (Formerly Institute of Lassa Fever Research and Control), Irrua Specialist Teaching Hospital, Irrua, Edo State Nigeria; 3 Pan-African Network For Rapid Research, Response, Relief and Preparedness for Infectious Disease Epidemics (PANDORA-ID-NET), Institute of Viral Hemorrhagic Fevers and Emerging Pathogens (Formerly Institute of Lassa Fever Research and Control), Irrua Specialist Teaching Hospital, Irrua, Nigeria; 4 Institute of Viral Hemorrhagic Fevers and Emerging Pathogens (formerly Institute of Lassa Fever Research and Control) and Department of Internal Medicine, Irrua Specialist Teaching Hospital, Irrua, Edo State Nigeria; 5 Infectious Disease Unit, Department of Internal Medicine, Irrua Specialist Teaching Hospital, Irrua, Edo State Nigeria; 6 Institute of Viral Hemorrhagic Fevers and Emerging Pathogens (formerly Institute of Lassa Fever Research and Control) and Department of Obstetrics and Gynecology, Irrua Specialist Teaching Hospital, Irrua, Edo State Nigeria; 7 Department of Obstetrics and Gynecology, Irrua Specialist Teaching Hospital, Irrua, Edo State Nigeria; 8 Department of Medical Microbiology, Irrua Specialist Teaching Hospital, Irrua, Nigeria; 9 Dermatology and Venereology Unit, Department of Internal Medicine, Irrua Specialist Teaching Hospital, Irrua, Nigeria; 10 Pan-African Network For Rapid Research, Response, Relief and Preparedness for Infectious Disease Epidemics (PANDORA-ID-NET) and Department of Obstetrics and Gynecology, Irrua Specialist Teaching Hospital, Irrua, Nigeria

**Keywords:** Mpox, Irrua, Genital presentation, Atypical presentations, Nodulopustular morphology

## Abstract

**Background:**

Mpox, previously known as monkeypox, -is an orthopoxvirus infection of the skin and previously a public health emergency of international concern. It reemerged in Nigeria over 5 years ago and has since spread to other parts of the world. This is a case report of a confirmed patient who was managed at Irrua Specialist Teaching Hospital, Irrua, Edo State, Nigeria before the global surge. This report shows peculiar differences from previous patients managed at the same center in terms of the relatively prolonged eruptive phase, possible seasonal occurrence of mpox in the community, and some traditional care for mpox and skin rashes. It also corroborates previous reports of possible sexual transmission of mpox in Nigeria before the report from the global outbreak.

**Case presentation:**

The patient is a 30-year-old Nigerian male artisan with a 2-month history of raised rashes on the body that started on the genitals then involved other parts of the body. There was history of sore throat and unprotected sex with a female partner with similar rash whose other sexual history could not be ascertained. There was also history of “seasonal” rash in his village for about 7 years prior to his symptoms. Examination showed multiple vesicles and some nodules (ulcerating, healing, and healed) on the face, trunk, limbs, gluteal region, scrotum, palms, and sole, an almost circumferential penile ulcer, and lymphadenopathy. Polymerase chain reaction skin samples sent for mpox returned positive, while retroviral and coronavirus disease 2019 screenings were negative. He was managed in isolation while contact tracing in the affected community was initiated.

**Conclusion:**

Atypical presentations of mpox, as managed in Irrua before the global surge, emphasize the varied spectrum of presentations (typical and atypical) in Nigeria. Therefore, there is a need for a higher index of suspicion for the uncommon presentations which will strengthen case recognition, case management, and community-based interventions as well as surveillance in the prevention and control of mpox in Irrua, its environs, Nigeria, and the world.

## Introduction

Mpox infection is a cutaneous infection caused by the mpox virus, which belongs to the Poxviridae family and *Orthopoxvirus* genus [1, 2]. It was previously designated as a public health emergency of international concern (PHEIC) by the World Health Organization (WHO) and has also been described as a potential weapon of bioterrorism and a potentially dangerous zoonosis [1–4]. It is opined that the cessation of smallpox vaccination over 40 years ago may have contributed to the resurgence of mpox, most likely because of reduced cross-immunity from the antibodies to smallpox [5]. This, in addition to changing behavioral patterns and environmental factors, may also explain the variation in the clinical presentation of mpox.

Mpox in Nigeria has shown a progressively increased burden with a peak in 2022, while a total of 988 confirmed cases have been reported from September 2017 to 1 January 2023 [6]. It has varying epidemiological characteristics across age groups, sex, morbidity, mortality, seasonality of infection, and regions of the country [1, 2, 5]. It is most common in South South, Nigeria, although the 2022 reports have shown a rising trend in other geopolitical zones [6]. As of June 2022, a total of 1715 cases (1636 suspected, 79 confirmed) and 73 deaths (CFR 4.3%) of mpox were reported in Africa (eight endemic and two nonendemic nations) according to Africa Centers for Disease Control and Prevention (CDC) [7]. Furthermore, with a double burden of the coronavirus disease 2019 (COVID-19) pandemic (February 2020 to June 2022), 12,141 cases and 363 deaths (CFR 3%) of mpox were documented [7]. The World Health Organization (WHO) record shows a decreased trend with a total of 87,972 confirmed cases with 147 deaths from 112 countries as of 19 June 2023 [8].

With reported outbreaks within and outside Nigeria, there is an urgent need for intensified surveillance. This report is aimed at documenting the uncommon presentation of a confirmed patients managed in a rural tertiary hospital in South South, Nigeria prior to the reports of atypical cases in nonendemic nations [7, 9]. It also aims to show some community based risks, perceptions, and care in resource-poor settings. This we hope will engender more renewed efforts at holistic and prompt diagnosis and management.

## Case presentation

A 30-year-old Nigerian male artisan who presented at the emergency room with a 2-month history of progressively raised rashes on the body that started on the genitals with onward involvement of the trunk, limbs, and face. There was a history of sore throat, painful penile ulcer, and swelling of the groin (lymphadenopathy) but no fever, body itching, or headache. He had unprotected sex with a partner who had itching, but the history of raised rashes could not be ascertained. There has been a history of similar “seasonal” rashes among his family members and close relations in his village that had spanned over 7 years. He had applied the traditional “white chalk” to the lesion without any improvement, thus necessitating his presentation to our center for optimal care.

**Fig. 1:**
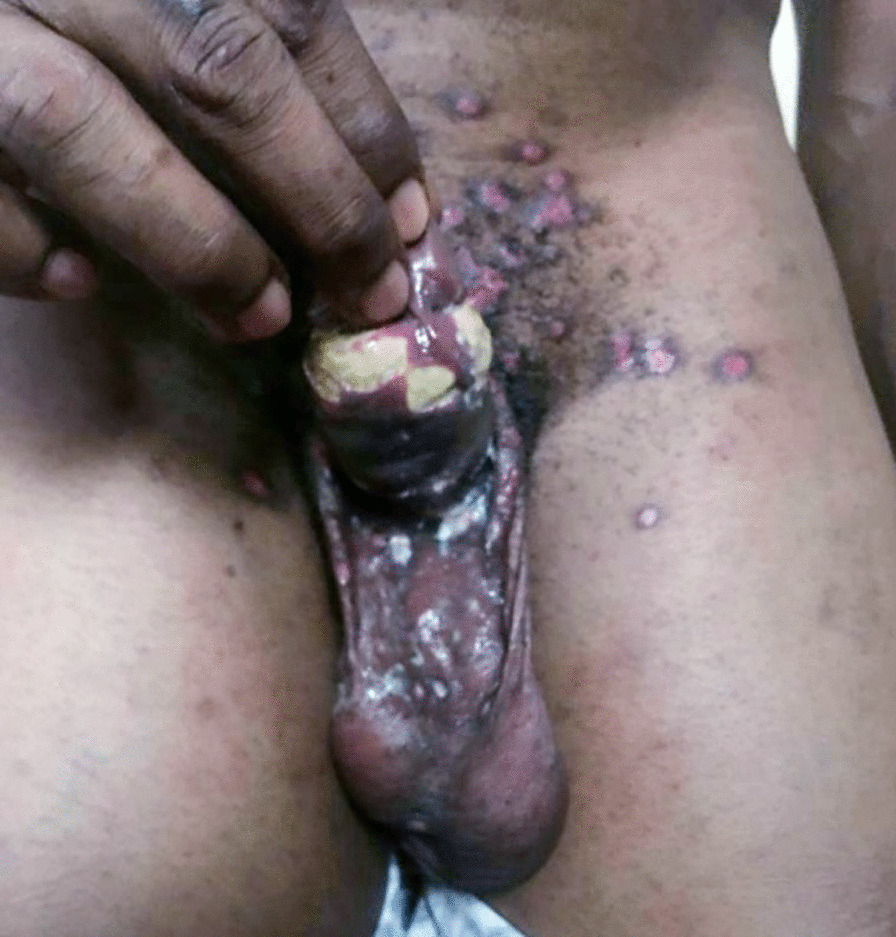
Penile ulcers with seropurulent discharge, sloughs, and surrounding erythematous healing scars around the phallus, suprapubic region, and superomedial part of the thighs

**Fig. 2:**
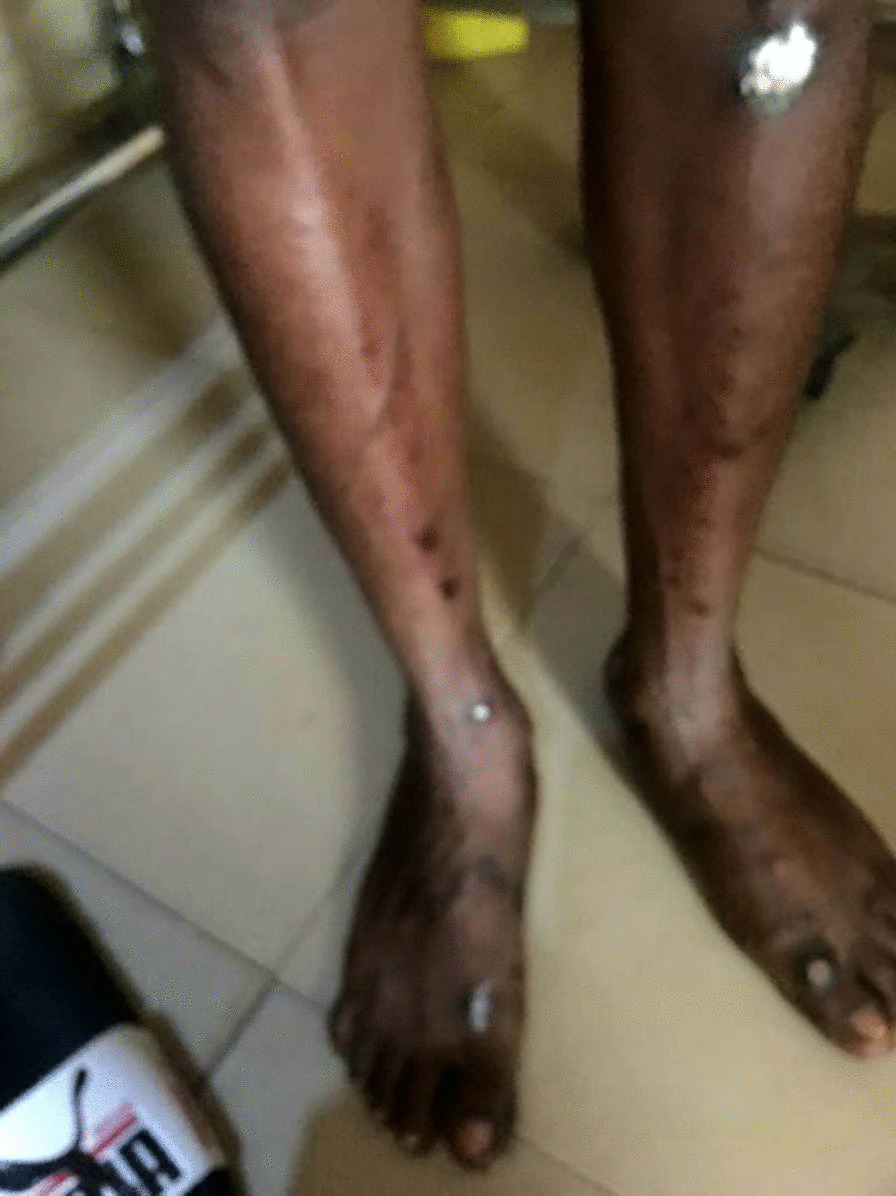
Healing scars covered with traditional white chalk and postinflammatory hyperpigmented macules and a few patches on the lower legs and feet

**Fig. 3:**
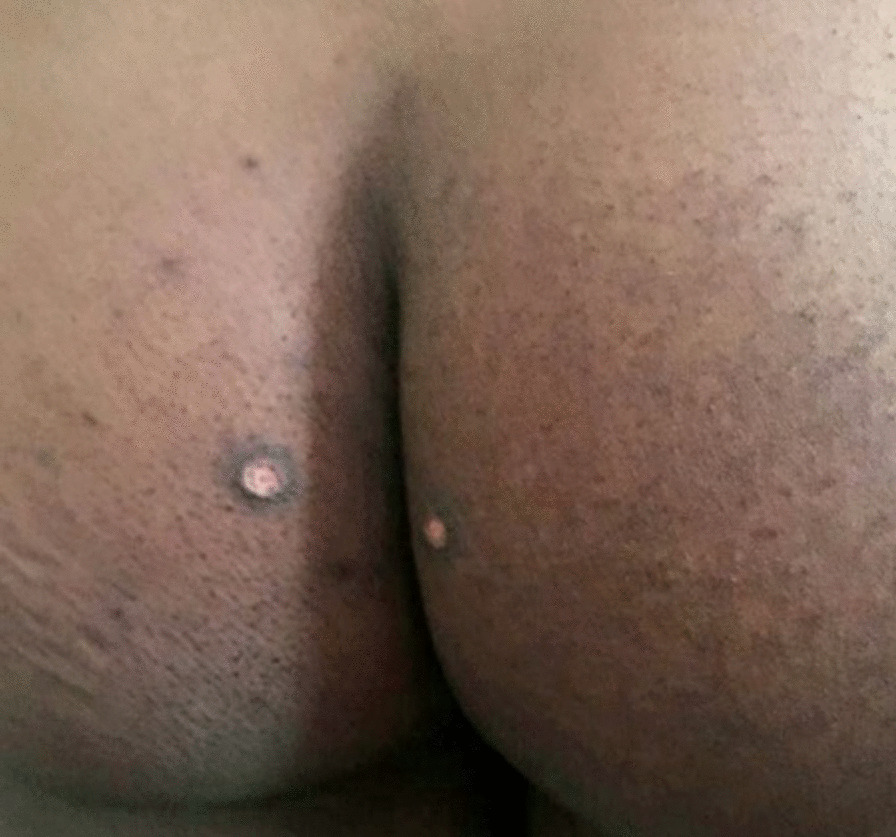
Healing scars on the gluteal region with some surrounding postinflammatory hyperpigmented macules

**Fig. 4:**
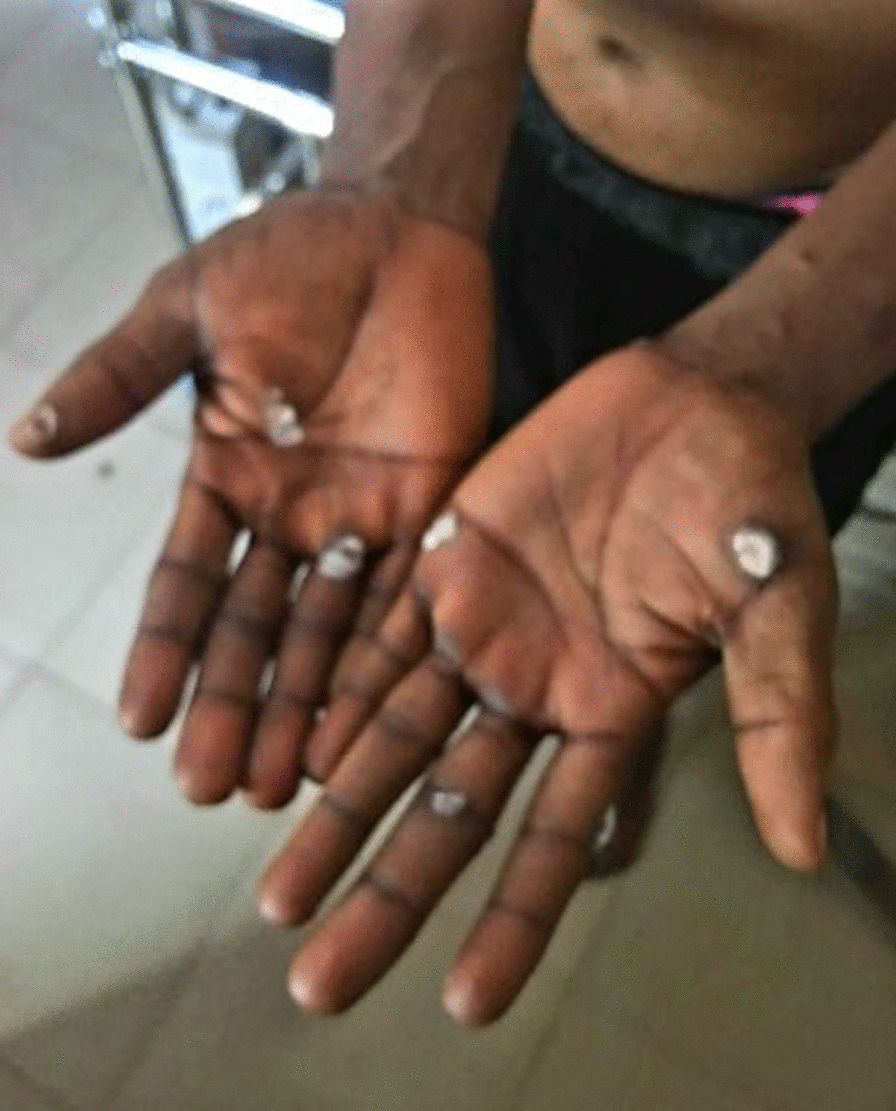
Healing scars on from rashes involving the palms with traditional white chalk having been applied by patient before presentation

Examination showed ruptured vesicles and some nodules (ulcerating, healing, and healed) on the suprapubic area, scrotum, face, trunk, limbs, gluteal region, palms, and sole (Figs. 1, 2, 3, 4) with an almost circumferential ulcer with slough involving the penile sulcus exuding seropurulent discharge (Fig. 1). There was submandibular and inguinal lymphadenopathy and reduced breath sounds on the left lower lung zone while other systems were normal.

Monkeypox was considered. Skin samples sent for mpox PCR returned positive. Retroviral and COVID-19 screening were negative. The patient was placed on intravenous 1 g ceftriaxone every 12 hours, 500 mg metronidazole every 8 hours, normal saline cleaning of the ulcers twice daily, and 800 mg acyclovir tablets five times daily that were well tolerated with no adverse effects. Contact tracing with a visit to his community for active case identification and management was promptly commenced in collaboration with the disease surveillance notification officer and the hospital public health team. Clinical conditions improved in isolation with satisfactory outcome on discharge and follow-up.

## Discussion

Mpox is an orthopoxvirus infection in both endemic and nonendemic parts of the world [1, 2]. Previously, the clades (mpox variants or subtypes) were called the Central Africa clade and the West African clade, but concerns about nondiscriminatory classification were raised with subsequent reclassification to clade I, IIa, and IIb [10]. Clades I and IIa correspond those common in Central African and West African, respectively, while clade IIb corresponds to those in previously nonendemic areas. Clade IIa has less severity than clade I. Mpox has similarities in morphology with some other viral skin infections such as the varicella zoster virus, smallpox, other poxviruses, scabies, and measles among others [11, 12]. The reservoirs for mpox is not yet known, although some reports have shown the virus isolated in rope squirrels and sooty mangabey monkeys with antibodies from other rodents, prairie dogs, and giant rats among others [13, 14].

The temporal profile of the viral infection often gives it away as the rash is reported to start from the head downwards, although this is contrary to the findings in this index patient whose rashes started in the genital area. Findings from other Nigerian works showed facial onset of rash and vesiculopustular rashes as opposed to the initial genital onset, human-to-human transmission, and some nodular findings as in this report [1, 2, 15]. This patient presented with healing ulcerated nodules, which is different from the predominant vesiculopustular presentations previously reported [1, 2]. Nodular presentations with a history of sexual contact had also been reported by Echekwube *et al.* in North Central, Nigeria [16]. This possibly suggests that, in addition to the vesiculopustular morphology, there is also nodulopustular morphology of mpox that is possibly underreported. More intentional review of the lesions with attention to the characteristic morphology will provide more data on the nodulopustular presentation. The findings of fever, pruritus, headache, and lymphadenopathy are similar findings to those from the neighboring state [2, 17]. Reports from the Nigeria Centre for Disease Control and Prevention (NCDC) also confirms these symptoms and signs [2].

Monkeypox usually begins with a flu-like illness and swelling of the lymph nodes as evident in this patient [1, 2, 7]. Human transmission is from prolonged contact with rashes or body secretions from an infected person, a fact that is accentuated by the history of unprotected sex before the onset of the rash in this patient. This then suggests the possibility of mpox being a “sexually transmitted infection” through the close contact during coitus as well as a possible droplet infection. However, appropriately powered studies are needed to establish this.

This patient presented with genital ulcers at a time it was uncommon and hardly reported in nonendemic regions of the world [7, 9]. However, genital ulcers as a presentation has previously been reported in a Bayelsa report as well as by Ogoina *et al.* [15, 17] Furthermore, it is also noteworthy to state that a previous documentation by Nigeria Centre for Disease and Control and Prevention (NCDC) had previously stated that mpox can affect any parts of the body [2].

Comorbidities are commonly reported in association with mpox infection and their impacts on the prognosis and outcome. Previous reports have also documented associated comorbidities such as pharyngotonsillitis and HIV coinfection, among others, with varying outcomes [2, 7, 9]. This patient presented with a chest infection and a documented complication of mpox, although screening for human immunodeficiency virus (HIV) and COVID-19 turned out negative.

Mpox has also been shown to have a progressive change in the age groups affected in over 50 years of documentation of its existence in human beings [5]. There is a relative increase in the decades of presentation—the thirties in this patient versus the twenties between 2010 and 2019 as previously reported. This change may give credence to the progressive instability of the viral pathogenic tropisms (host, tissue, and cell) coupled with the possible lack of antibodies from smallpox vaccination in this patient’s age group [5]. Since smallpox vaccination ended in 1980, it is likely that cross-protection from smallpox may be lacking in this age group, thereby causing immunologic naivety and increased susceptibility [5].

Community spread cannot be rejected within the context of mpox, especially bearing the direct contact and possible droplet route of infection in this context. This patient also disclosed a history of a similar rash in his village with a “seasonal” occurrence of the rash such that the villagers use “white powder” and “antiseptics” with acclaimed resolution. The knowledge of the nonfatal outcome of the clade in this environment may have also affected the health-seeking behavior in this community. Prompt surveillance (active and genomic) may help establish the epidemiologic pattern and clinical characteristics as well as determine the possible clades that may have been in this community [2, 5].

The community spread may have been due to the progressive encroachment of human activities on wildlife, increased intake of meat from wildlife (also known as “bush meat” in Nigeria), immunosuppression, and rapid urbanization. The syndemic of mpox and the COVID-19 pandemic has also been reported to have impacted the risk of transmission of mpox. The concentration of most resources on COVID-19 care, its associated job loss, lockdowns, and reported poverty may have also necessitated more engagement in wildlife activities that increase the risk of mpox [18, 19]. Furthermore, Tambo *et al.* had described mpox as a poverty-related disease bearing the many socioeconomic clusters that increase the human–rodent interface [18].

The communal “seasonal” rash in patient’s community, if confirmed to be mpox-related, may suggest the likelihood of some level of herd immunity. This may also give an explanation to the possible community perception and associated health-seeking behavior in resource-poor settings where mpox is yet to be confirmed. More information on the transmission (typical, possible asymptomatic, and perinatal transmission), genomics, and various diagnostic challenges will strengthen surveillance and risk-communication strategies that will ensure control and eradication of mpox [11, 12, 20–24].

The strength of this study lies in the information that this work adds to the body of knowledge such as the relatively prolonged eruptive phase of 2 months prior to presentation and the traditional and culture-based practices in the care of patients with mpox and skin rashes. Furthermore, it suggests the possible transmission of mpox within the community. Bearing the limitations of case reports, there is a need for appropriate studies for critical appraisal of the findings from this report.

## Conclusion

Mpox has both typical and atypical presentations. The peculiarity of duration of rash, clinical features, “seasonal” occurrence of a similar rash, as well as the knowledge on the culture based care in this patient’s community should be considered during clinical evaluation. Therefore, it is expedient to have a higher index of suspicion among health workers and more public health surveillance, sustained community education, and health promotion on mpox infection in Irrua, its environs, and Nigeria. Some culture-based practices in the care of mpox call for appropriate scientific work.

## Data Availability

The datasets supporting the conclusions of this article are included within the article.
